# ^1^H-NMR and MALDI-TOF MS as metabolomic quality control tests to classify platelet derived medium additives for GMP compliant cell expansion procedures

**DOI:** 10.1371/journal.pone.0203048

**Published:** 2018-09-06

**Authors:** Francesco Agostini, Marta Ruzza, Davide Corpillo, Luca Biondi, Elena Acquadro, Barbara Canepa, Alessandra Viale, Monica Battiston, Fabrizio Serra, Silvio Aime, Mario Mazzucato

**Affiliations:** 1 Stem Cell Unit, Department of Translational Research, CRO Aviano National Cancer Institute, Aviano (PN), Italy; 2 GEMFORLAB SrL, Colleretto Giacosa (TO), Italy; 3 BRACCO Imaging Italia Srl, Milano, Italy; 4 Molecular Imaging Center, Department of Molecular Biotechnologies & Health Sciences, University of Torino, Torino, Italy; 5 Clinical and Experimental Onco-Hematology Unit, Department of Translational Research, CRO Aviano National Cancer Institute, Aviano (PN), Italy; Monash University, AUSTRALIA

## Abstract

**Introduction:**

*Ex vivo* cell expansion under Good Manufacturing Practice (GMP) guidelines can be performed using medium additives containing human growth factors from platelets. These products can differently affect proliferation of adipose mesenchymal stromal stem cells (ASC). Qualification of medium additive performance is required for validation under GMP regulations: assessment of growth factor concentrations is not sufficient to predict the biological activity of the product batch. Proton nuclear magnetic resonance spectrometry (^1^H-NMR) and matrix-assisted laser desorption/ionization time of flight mass spectroscopy (MALDI-TOF MS) provide wide molecular characterization of samples.

**Aims:**

We aimed to assess if ^1^H-NMR and MALDI-TOF MS techniques can be used as quality control test potentially predicting the impact of a medium additive on cell proliferation.

**Methods:**

We tested the impact on ASC growth rate (cell proliferation assessment and cell morphology analysis) of four medium additives, obtained by different methods from human platelet apheresis product. In order to classify each medium additive, we evaluated growth factor concentrations and spectra obtained by ^1^H-NMR and by MALDI-TOF MS.

**Results:**

Medium additive obtained by CaCl_2_ activation of platelet rich products induced higher proliferation rate *vs* additive derived from platelet depleted ones. Additives obtained by freeze-and-thaw methods weakly induced ASC proliferation. As expected, principal component analysis of growth factor concentrations did not unravel specific biochemical features characterizing medium additives in relation with their biological activity. Otherwise, while ^1^H-NMR showed a partial resolution capacity, analysis of MALDI-TOF MS spectra allowed unambiguous distinction between the medium additives we used to differently stimulate cell growth *in vitro*.

**Discussion:**

MALDI-TOF and, despite limitations, ^1^H-NMR are promising cost effective and reliable quality controls to classify the potential of a medium additive to promote ASC growth. This can represent, after further investigations and appropriate validation, a significant advantage for GMP compliant manufacturing of advanced cell therapy products.

## Introduction

*Ex vivo* cell expansion is a fundamental procedure to obtain an advanced cell therapy (ACT) product: Good Manufacturing Practice (GMP) guidelines recommend to avoid animal derived serum as source of growth factors promoting cell proliferation *in vitro* [1]. In a previous work [2] we described our method to obtain a platelet releasate that we defined as supernatant rich in growth factors (SRGF): the mixture was manufactured adding CaCl_2_ to human platelet rich plasma (PRP) derived from apheresis product. In the same work [2], we attempted to characterize the amount of growth factors released by platelets. In a previously published work, we demonstrated that such GMP compliant medium additive can efficiently stimulate stromal cell proliferation in culture [3]. Moreover, in a recent paper [4] we demonstrated that SRGF can strongly promote growth of adipose mesenchymal stromal stem cells (ASC) by direct analysis of cell proliferation and evaluating cell morphology. Rapidly dividing mesenchymal stem cells are, in fact, known to be elongated and spindle shaped [5,6]. Interindividual differences between platelet donors could affect the capability to stimulate cell growth *in vitro* [7,8]: we demonstrated that, pooling together n = 16 SRGF products from single donors, standardized batches of medium additive can be obtained for applications in academic GMP facilities [4,9]. Nevertheless, we previously demonstrated that also the production method can affect medium additive capacity to stimulate cell growth in culture [10].Growth factors can, in fact, be extracted from platelets treating PRP by the so called freeze-and-thaw method [2,11], and we previously demonstrated that medium additives obtained by recalcification of PRP can promote a faster cell proliferation rate [10]. Especially when dealing with in-house manufactured medium additives, quality controls are needed also to assess and validate the capacity of the product to stimulate cell proliferation *in vitro* [9]. Direct evaluation of cell proliferation rate induced by different lots of medium additives is a long lasting test representing a significant workload in the production process. Growth factor concentration analysis is frequently used to characterize composition of platelet derived products with possible application as medium additives for cell expansion [2,12–15].Several growth factors released by platelets can affect cell proliferation rate by separate pathways [16]: thus, defined cytokines univocally affecting cell proliferation rate were not identified and validated as specific markers. Moreover, we previously demonstrated that, assessing growth factor concentrations, SRGF could not be clearly distinguished from freeze-and-thaw manufactured products [2,10]. Then, independently from the manufacturing method, a quick and cost effective test potentially predicting the impact of a medium additive on cell growth rate would represent a significant achievement improving both production and quality control processes.

Proton nuclear magnetic resonance spectrometry (^1^H-NMR) as well as matrix-assisted laser desorption/ionization time of flight mass spectroscopy (MALDI-TOF MS) allow, in a single analytical session, the simultaneous detection of several chemical compounds or functional groups [17]. Sample analysis by ^1^H-NMR and MALDI-TOF MS is relatively quick and economically effective and technological improvements are rapidly increasing sensitivity of such approaches [18–20]. In a previous work, biochemical alterations occurring along with storage of platelet concentrates obtained by apheresis were quantitatively defined by a mass spectrometry approach [21].

In this paper we aimed to verify whether the analysis of growth factor concentrations or ^1^H-NMR and MALDI-TOF MS spectra evaluation can identify medium additives differently promoting cell growth in culture. To create a challenging experimental model we manufactured, from human PRP, four kinds of medium additives sharing several biochemical features. Manufacturing protocols were based on CaCl_2_ addition and on the freeze and thaw method. We then directly compared the capacity of such medium additives to stimulate growth of ASC [22] in culture. Thereafter, in the attempt to discriminate differences characterizing each medium additive, we analyzed selected growth factor concentrations and composition of spectra obtained by ^1^H-NMR and MALDI-TOF MS.

## Materials and methods

### Study design

This work was aimed to define innovative, time and cost effective quality control tests potentially predicting the capacity of a GMP compliant medium additive to stimulate *in vitro* cell growth for ACT. Applying four separate production protocols to the same source of PRP, i.e. the platelet apheresis product obtained from healthy donors (n = 20), we manufactured 4 kinds of medium additives (n = 80 specimens). The impact of medium additives (pools of n = 20 single donor derived products) on *in vitro* cell growth was analyzed by a luminescence based approach and evaluating cell morphology. Then, in single donor derived medium additives, we measured and analyzed a) concentrations of selected growth factors, b) ^1^H-NMR and MALDI-TOF MS spectra. Results were analyzed by statistical tests and algorithms in the attempt to highlight differences between groups of single donor samples of the four medium additives.

### Medium additive preparation

The experimental protocol was approved by the Ethics Committee of the CRO Aviano National Cancer Institute (protocol number: CRO-2016-30) and it complied with the Declaration of Helsinki (2004). Single donor PRP samples were collected at CRO Aviano National Cancer Institute from washouts of leukocyte depletion filters intended for disposal, taken from platelet apheresis collection-kits (Haemonetics MCS+ System; Haemonetics, Signy-Centre, Switzerland) after donation from healthy donors. Citrate Dextrose Solution A (Haemonetics; Braintree, MA, USA) was used as anticoagulant during platelet collection. Labels and/or information regarding personal data of donors were removed before manipulation of the product in the processing facility. Briefly, after bags containing the apheretic product were separated from the kit, leukocyte depletion filter was removed by upstream and downstream triple tube welding, thus maintaining product sterility. Under a sterile biological safety hood, the filtered apheresis product was transferred to a sterile tube (Becton, Dickinson and Company - BD, Franklin Lakes, NJ, US). Mean platelet content in PRP was 1.1 ± 0.2 (x 10^6^ platelets/microL). In compliance with the Italian legislative decree of 2^nd^ of November 2015 “Disposizioni relative ai requisiti di qualità e sicurezza del sangue e degli emocomponenti” [23] the product pH evaluated after collection was greater than 6.4. Platelet apheresis product from each single healthy donor (n = 20) was divided in 4 separate samples and it was treated as previously published with modifications [2] to manufacture the different medium additives. Additive A (recalcified PRP) was obtained adding CaCl_2_ (4.4% vol/vol) to PRP in order to trigger calcium dependent coagulation cascade via endogenous thrombin generation. After 1 hour incubation at 37°C, formed clot was separated from supernatant by centrifugation at 2300 X g for 10 minutes at room temperature. To produce additive B (recalcified platelet poor plasma - PPP) a separate PRP aliquot was centrifuged (1600 X g for 10 minutes) in order to generate PPP. Collected supernatant was added with CaCl_2_ (4.4% vol/vol) and after 1 hour incubation at 37°C, clot was separated from supernatant by centrifugation. To obtain medium additive C (freeze and thaw, plasma depleted platelets) PRP was centrifuged (1600 X g for 10 minutes) and the supernatant was discarded. Original sample volume was reconstituted gently resuspending platelets in phosphate-buffered saline (PBS) solution. Plasma depleted platelets underwent three cycles of freezing in liquid nitrogen (-190°C for 2 h) and thawing at +40°C (30 min). After centrifugation, supernatant was collected. Medium additive D (freeze and thaw, PRP) was obtained performing three cycles of freezing in liquid nitrogen (-190°C for 2 h) and thawing at +40°C (30 min) on PRP. After centrifugation, supernatant was collected. pH was measured in obtained medium additives A, B, C and D: values were within the range between 6.7 and 7.0. Samples were stored at -80°C.

### Cell proliferation test

To test the potential of investigated medium additives to stimulate cell growth *in vitro*, we created batches of medium additives A (recalcified PRP), B (recalcified PPP), C (freeze and thaw, plasma depleted platelets) and D (freeze and thaw, PRP) pooling together aliquots (200 microL) of the relative product obtained from single donor platelet apheresis product (batch final volume: 4ml).

ASC were chosen as model of *in vitro* proliferating cells: the experimental protocol was approved by the Ethics Committee of the CRO Aviano National Cancer Institute (protocol number: CRO-2016-30) and it complied with the Declaration of Helsinki (2004). Cells were obtained as previously published [4] from n = 3 separate donors and they were seeded at passage P4 in 96-well plates (BD Biosciences, Bedford, MA; US) at 3000 cells/cm^2^ in complete Minimum Essential Medium Eagle - Alpha Modification (Alpha-MEM - Lonza; Basel, Switzerland)) containing 100 IU/ml of Penicillin, 100 microg/ml of Streptomycin (both from Sigma, St. Louis, MO; USA) and 5% (vol/vol)SRGF. After 4-6 hours, adherent cells were washed and medium was replaced with Alpha-MEM containing antibiotics and 5% (vol/vol) A, B, C and D (pools) each in four technical replicate wells. ASC were also cultured in presence of Alpha-MEM containing 10% (vol/vol) fetal bovine serum (FBS - Lonza) as control condition. Cell growth was assessed after 1, 4 and 7 days by a luminescence based commercially available kit (CellTiter-Glo®Luminescent - Cell Viability Assay; Promega, Madison, WI, US) using an Infinite® F200 (Tecan; Männedorf, Switzerland) as luminescence reader. To assess background, at each time point, luminescence values were taken in wells containing only cell growth media.

The impact of the different medium additives on cell proliferation was assessed also evaluating cell morphology. ASC were seeded in 24-well plates (BD Biosciences) at 3000 cells/cm^2^: after 4-6 hours, adherent cells were washed and medium was replaced with Alpha-MEM containing antibiotics and 5% (vol/vol) A, B, C, D (pools) and 10% (vol/vol) FBS, each in two technical replicate wells. After seven days exposure to experimental media, cell images were taken by phase contrast microscopy (Olympus CKX41, Olympus Italia Srl, Milano; Italy) and digital color camera (Motic; Hong Kong). Morphometric analysis was performed taking advantage of Motic Images Plus 2.0 software® after appropriate calibration. At least 40 cells expanded by each different medium additive were manually analyzed. After calibration, cell major and minor axes were measured using appropriate software tools following manufacturer’s protocol. Major-to-minor cell axis ratio was then calculated.

### Quantitative analysis of growth factors

As previously published [2] concentrations of growth factors were measured in medium additives obtained from single donor PRP by enzyme-linked immunosorbent assay (ELISA) using commercially available kits (Quantikine ELISA kit; R&D Systems, Minneapolis, MN, USA). The following growth factors were assayed: platelet-derived growth factor (PDGF)-AA (kit catalogue n.: DAA00B); PDGF-AB (kit catalogue n.: DHD00C), PDGF-BB (kit catalogue n.: DBB00), transforming growth factor-beta 1(TGF-b; kit catalogue n.: DB100B), fibroblast growth factor basic (FGF; kit catalogue n.: DFB50), Epidermal growth factor (EGF; kit catalogue n.: DEG00), insulin-like growth factor 1 (IGF-1; kit catalogue n.: DG100) and vascular endothelial growth factor (VEGF; kit catalogue n.: DVE00). The mean kit intra-assay coefficient of variation was 4.3% (min: 2.4%; max: 8.32%). The mean kit inter-assay coefficient of variation was 7.9% (min: 5.0%; max: 11.5%). All used ELISA kit were specific for the tested analyte. For technical reasons, growth factors were analyzed only in samples derived from 7 out of the 20 PRP from healthy donors involved in this study. Even though analytical properties of multiplex immunoassays are growing, in this work we chose singleplex ELISA as such robust analytic approach is normally adopted for diagnostic purposes.

### ^1^H-NMR analysis

Samples were thawed at room temperature and diluted (1:1 ratio) with PBS at pH 7.4 containing 10% D_2_O and 5 mM trimethylsilylpropanoic acid.

All ^1^H-NMR experiments were performed at 298 K on a Bruker Avance DRX-600 spectrometer (Bruker, Karlsruhe, Germany) operating at 600.13 MHz for proton and equipped with a 5-mm BBI ^1^H/2H-BB-Z-GRD probe. For each sample standard 1D spectroscopy, relaxation-edited, and diffusion-edited pulse sequences were acquired in order to monitor different groups of metabolites.

1D proton spectra were acquired using the NOESYPR1D (1D Nuclear Overhauser effect spectroscopy with water pre-saturation) pulse sequence, with a relaxation delay of 5 s and a mixing time of 10 ms. Water suppression was achieved by pre-saturation during the relaxation delay and mixing time. Each spectrum consisted of 64 free induction decays collected into 96K complex data points with a spectral width of 17985.61 Hz and an acquisition time of 2.73 s.

1D relaxation-edited ^1^H-NMR spectra were acquired using the water suppressed Carr-Purcell-Meiboom-Gill (CPMG) spin echo pulse sequence with a relaxation delay of 5 s and a total echo time of 38.4 ms in order to attenuate broad signals from proteins and lipoproteins. 64 FIDs were collected into 64K complex data points.

Diffusion-edited spectra were acquired using a pulse sequence with bipolar gradients and the LED scheme, with relaxation delay = 5 s, number of FIDs = 64, 32K complex data points, diffusion delay = 120 ms and pulsed-field gradient = 1.5 ms.

### ^1^H-NMR spectral processing

Prior to Fourier transformation, the FIDs were zero-filled to 64K points and multiplied by an exponential line-broadening function of 0.3 Hz. The 1D spectra were manually phased, baseline corrected and the chemical shifts referenced internally to the Lactate signal at delta 1.31 ppm, using dedicated Topspin™ software (Bruker, Karlsruhe, Germany).

### MALDI-TOF MS analysis

Samples were thawed at room temperature and 5 microL of each sample were processed with ClinProt MB HIC 8 profiling kit according to manufacturer's instructions. The elution volume used to recover each sample from magnetic beads was 10 μI. 3.5 microL of eluate were then mixed 1:1 with a saturated solution of sinapinic acid and spotted onto a MALDI target plate.

Mass spectra analyses were performed by using a MALDI-TOF detector (Ultraflex II instrument; Bruker Daltonics). Mass spectra were acquired in a range between 2,000 and 23,000 m/z, in positive linear mode.

### MALDI-TOF mass spectra processing

Mass spectra were processed by a dedicated software (ClinProTools, Bruker Daltonics) that allows visualization and comparison of large data-sets, analysis of peak areas and the subsequent creation of models for the recognition of peptide and protein profiles.

### Statistical analysis

#### Cell proliferation rate

Luminescence values are represented as mean±SEM. The impact of the different medium additive on mean luminescence values at day 7 as well as on major-to-minor axis ratio was analyzed by ANOVA for independent samples with Tukey’s honestly different significance with Bonferroni’s correction as *post hoc* test.

#### Growth factor concentrations (ELISA)

Growth factor concentrations data obtained by ELISA are represented as mean±SEM. For each separate growth factor, one-way ANOVA for independent samples was used to compare mean concentrations in the different medium additives. Tukey’s honestly different significance with Bonferroni’s correction was chosen as *post hoc* test. Moreover, growth factor concentration values, measured in each medium additive sample derived from single donor PRP were analyzed by Principal Component Analysis (PCA). The PCA creates a model that compares each sample considering the variance within a dataset and using a small number of actors (principal components). PCA of growth factor concentration data was performed by "R-software" (an open source statistical software for statistical computing and graphics) using the plotting system “ggplot2”.

#### ^1^H-NMR spectra

Each ^1^H-NMR spectrum was divided in small regions (0.01-0.05 ppm) called buckets, and each bucket integral was calculated. Then, all the buckets were compared to obtain the bucketing average: this was considered as the reference to be compared with the buckets of each single spectrum. The resulting numerical matrix was analyzed by PCA in order to compare the four different groups A (recalcified PRP), B (recalcified PPP), C (freeze and thaw, plasma depleted platelets) and D (freeze and thaw, PRP) of medium additive samples. PCA of ^1^H-NMR derived data was performed by the dedicated Amix software (Bruker, Karlsruhe, Germany) and the spectral region between 4.7 and 5.0 ppm was removed prior to statistical data analysis to avoid variability due to the residual water signal.

#### MALDI-TOF MS spectra

The MALDI-TOF MS spectra were processed by the dedicated ClinProTools software in order to compare the four different groups A (recalcified PRP), B (recalcified PPP), C (freeze and thaw, plasma depleted platelets) and D (freeze and thaw, PRP) of medium additive samples obtained from single PRP donors. Considering obtained results, the software generated three classification models (Genetic Algorithm, Quick Classifier and Support Vector Machine), based on separate algorithms.

## Results

This work was aimed to define innovative, time and cost effective quality control tests classifying the capacity of GMP compliant medium additives to stimulate cell growth *in vitro*. Applying four separate production protocols to the same source of platelet concentrates (i.e. PRP from platelet apheresis collected from healthy donors), we obtained 4 kinds of medium additives: A (recalcified PRP), B (recalcified PPP), C (freeze and thaw, plasma depleted platelets) and D (freeze and thaw, PRP).

**Fig 1:**
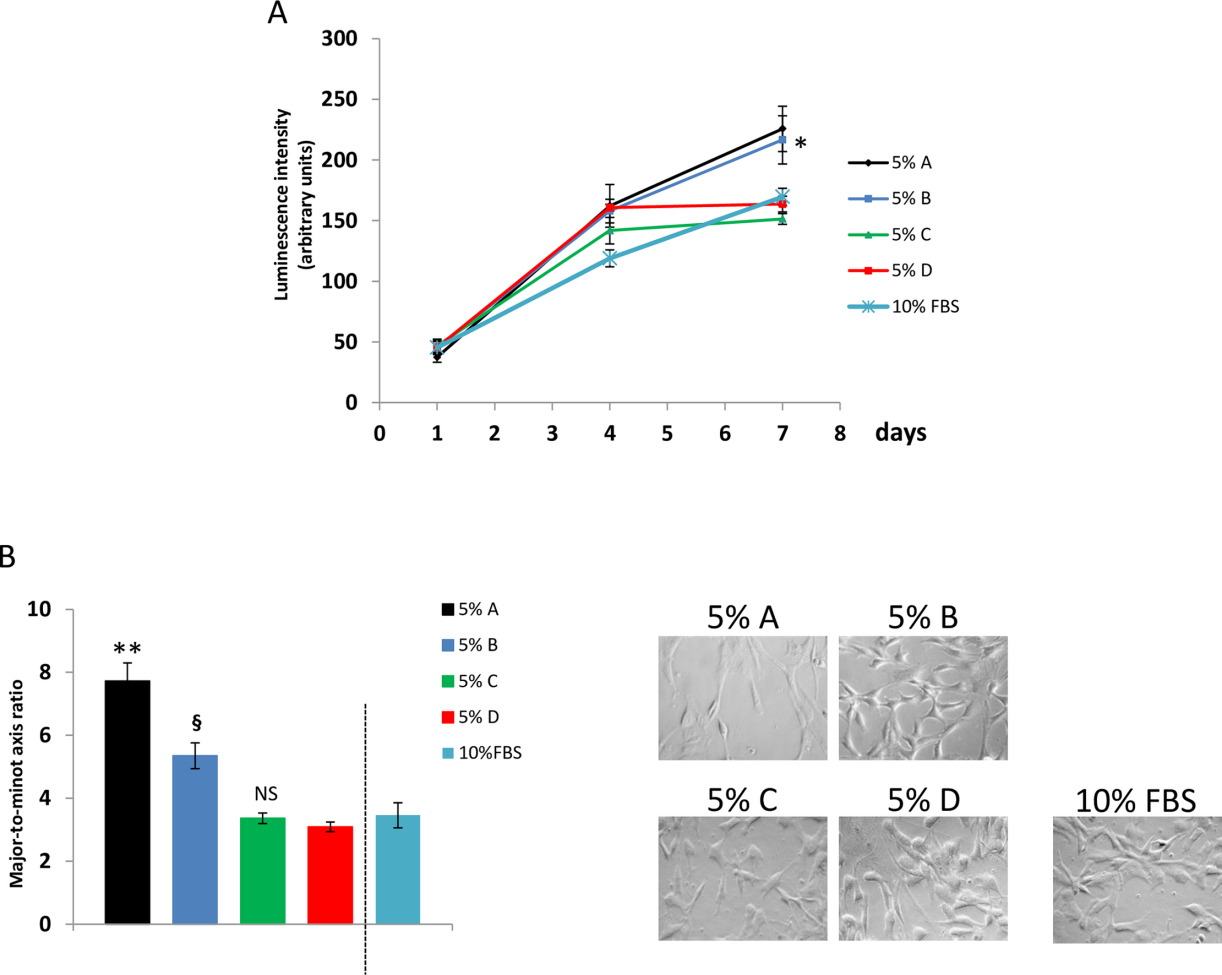
**Impact of medium additives A, B, C and D on proliferation rate of ASC**. (A) Cell growth test based on luminescence generated by living and proliferating cells. Results show that in presence of 5% medium additive A (recalcified PRP) and B (recalcified PPP) cell proliferation rate is higher, when compared to additives C (freeze and thaw, plasma depleted platelets) and D (freeze and thaw, PRP). (B) Mean values of major-to-minor axis ratios and representative images of ASC expanded in presence of the different additives. ASC expanded in presence of medium additives A and B were more elongated than ASC expanded in C and D. ASC expanded with additive A were more elongated than ASC cultured with additive B. Values of luminescence and of major-to-minor axis ratios obtained from cells expanded in presence of 10% FBS were shown as control condition. Considering such results, ASC expanded in presence of medium additive A showed higher proliferation rate when compared to additive B. When compared to medium additives A and B, ancillary products C and D weakly stimulated cell proliferation. ANOVA for independent samples was performed as statistical analysis (p<0.001) and Tukey’s honestly different significance with Bonferroni’s correction was chosen as *post hoc* test to compare the effects of the different medium additives.*, p<0.05 vs C and D; **, p<0.01 vs B, C and D; ^§^, p<0.05 vs C and D; NS vs D.

We assessed the impact of medium additives (as pools of n = 20 single donor derived products) on cell proliferation rate by a luminescence based approach and evaluating cell morphology. Results reported in Fig 1A show luminescence values reflecting the number of ASC cultured for 1, 4 and 7 days in presence of medium additives (pools) at the final concentration of 5%. ANOVA for independent measures showed that the number of expanded cells after 7 days in culture with 5% additives A (recalcified PRP) and B (recalcified PPP) was significantly (p<0.01) higher when compared to additives C (freeze and thaw, plasma depleted platelets) and D (freeze and thaw, PRP). Proliferation rate of cells expanded in 10% FBS was shown as control condition: luminescence values were aligned with those obtained using 5% additives C and D. Raw luminescence values collected to estimate ASC growth rate in presence of the different tested medium additives were reported in S1 File. Similarly, as displayed in Fig 1B, ASC expanded in presence of medium additives A and B were more elongated than ASC expanded in presence of additives C and D. In addition, ASC expanded with medium additive A (recalcified PRP) were shown to be more elongated than ASC cultured with medium additive B (recalcified PPP). Representative images of ASC expanded in presence of the different additives were reported in Fig 1B. Morphology of cells expanded in 10% FBS was evaluated as control condition: major-to-minor axis ratio of such cells was shown to be superimposable with related values measured in ASC expanded with 5% additives C and D. Major-to-minor-axis ratio values, collected to estimate ASC growth rate in presence of the different tested medium additives, were reported in S2 File.

### Growth factor concentrations

**Fig 2:**
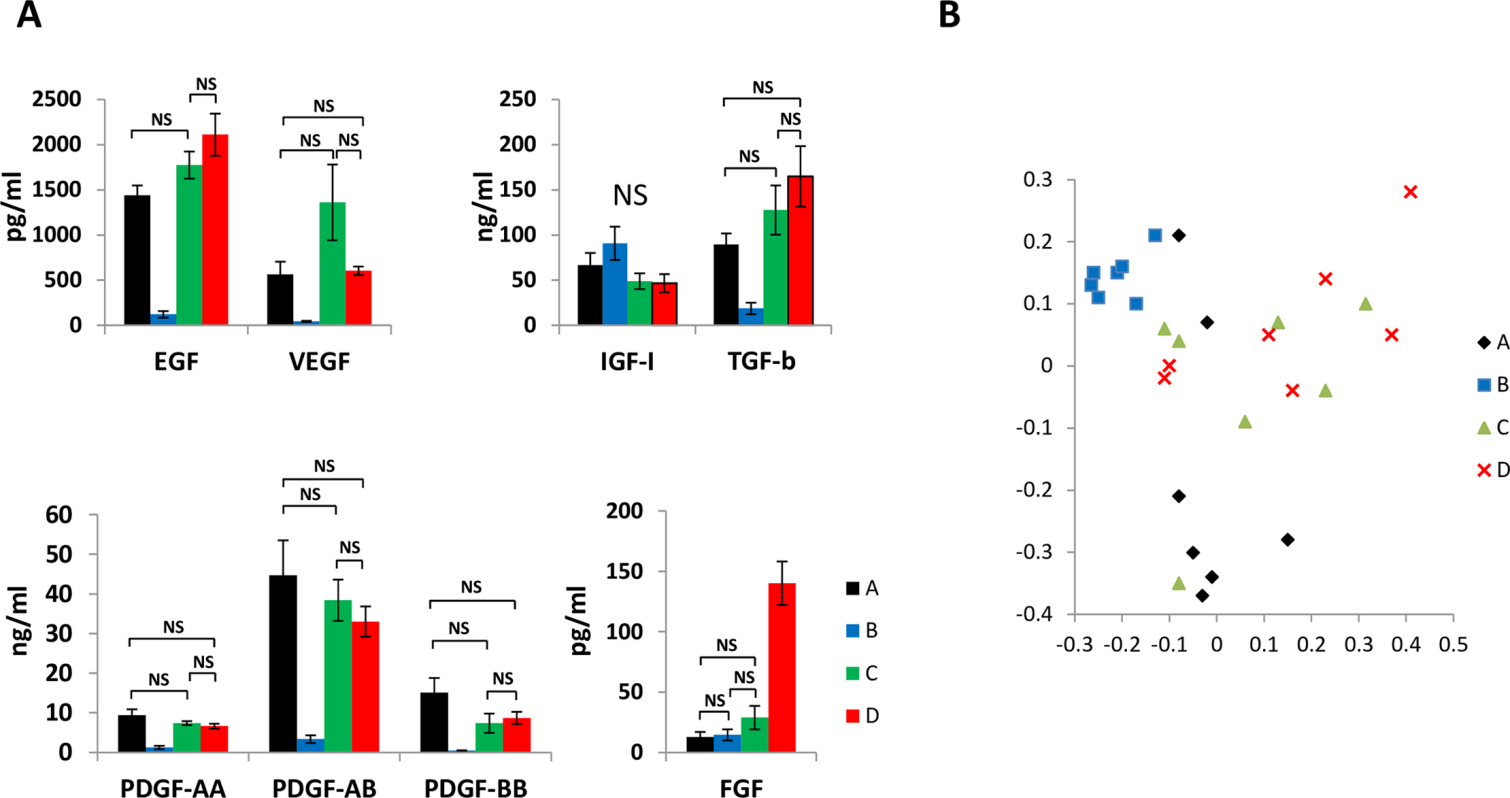
Analysis of growth factor concentration in different medium additives. (A) Concentrations of selected growth factors measured in samples of the four medium additives A (recalcified PRP) and B (recalcified PPP), C (freeze and thaw, plasma depleted platelets) and D (freeze and thaw, PRP). Statistical analysis was performed by one-way ANOVA for independent samples and Tukey’s honestly different significance and Bonferroni’s correction was chosen as *post hoc* test. Not significant (NS) differences were graphically indicated by solid lines in the different graphs. Concentration values analyzed within each growth factor displayed statistically significant (p<0.05) differences only when not connected by solid lines. (B) Graphical representation of PCA performed on concentration values of all growth factors evaluated in single donor samples of the different medium additives. Exception made for medium additive A, samples of the other medium additives could not be segregated in separate clusters.

In order to characterize medium additives we used in this paper, we measured concentrations of selected growth factors in different medium additives by ELISA (Fig 2A). Raw growth factor concentration values measured in samples of the different medium additives were reported in S3 File. IGF-I concentration was not differently modulated in the four medium additives. Concentrations of the other analyzed growth factors showed significant differences only when separately comparing selected couples of medium additives. Not significant concentration differences could be demonstrated when comparing two and even three possible couples of medium additives. Then, we could not identify one or more growth factors displaying significantly different concentration values when comparing all the analyzed medium additives within the same statistical analysis. Thereafter, we analyzed by PCA all single growth factor concentrations values measured in each sample derived from single donor products: results displayed in Fig 2B show that, exception made for medium additive A (recalcified PRP), samples of the other medium additives could not be segregated in separate non-overlapping clusters. Therefore, MALDI-TOF MS and ^1^H-NMR were tested as alternative approaches to unravel biochemical differences between the investigated human platelet derived medium additives.

### ^1^H-NMR spectroscopy

**Fig 3:**
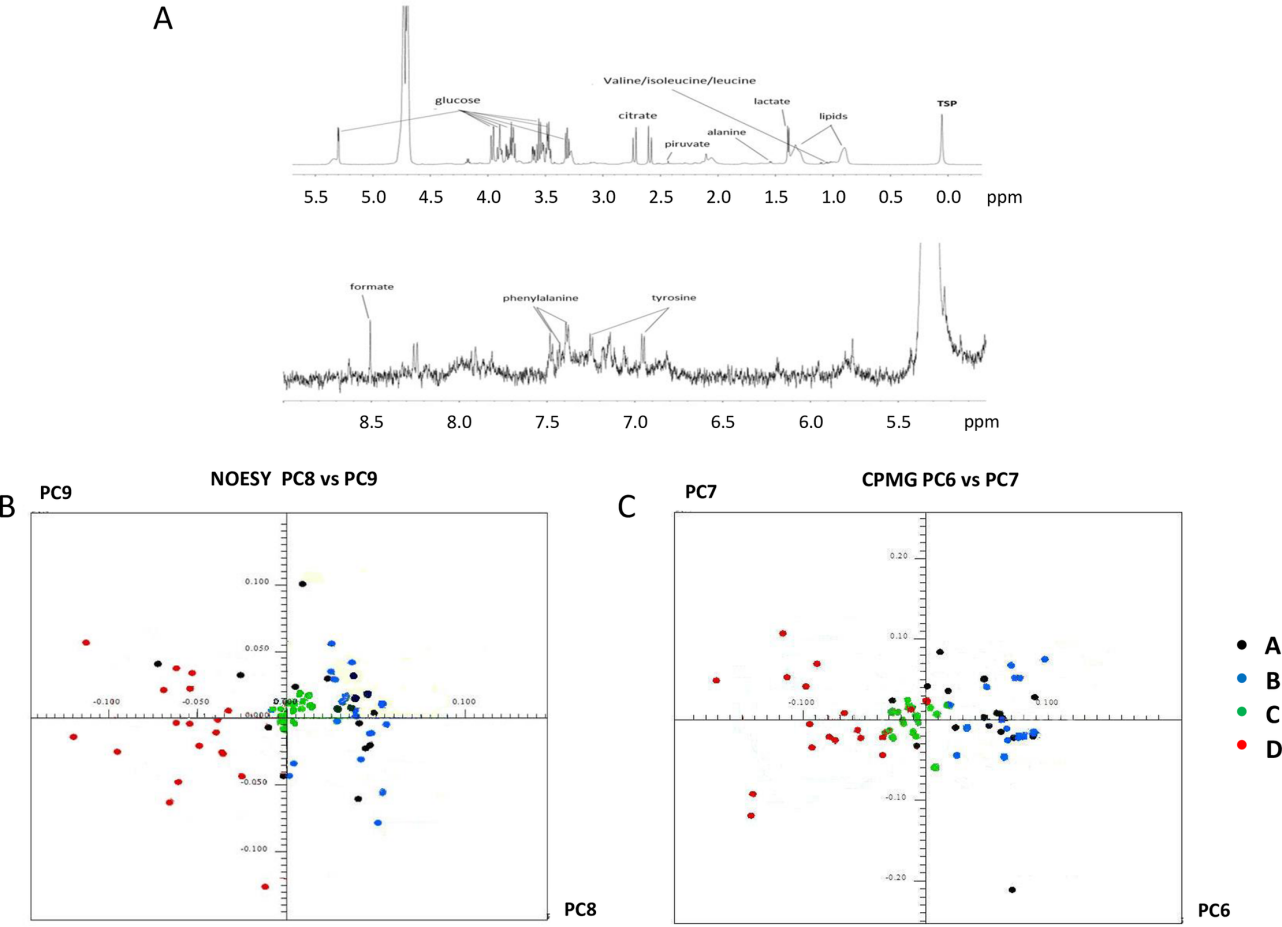
Sample analysis by ^1^H-NMR. (A) Representative ^1^H-NMR spectrum, with attribution of some of the observed peaks to known metabolites. All spectra were characterized by high reproducibility and consistency and no major variations among different samples could be identified. (B) PCA of NOESY spectra considering PC8 vs PC9. (C) PCA of CPMG spectra considering PC6 vs PC7. Clusters enclosing samples of medium additives B (recalcified PPP), C (freeze and thaw, plasma depleted platelets) and D (Freeze and thaw, PRP) were reciprocally segregated while the cluster related to samples of medium additive A (recalcified PRP) was scattered and partially overlapping with B and C.

A representative example of ^1^H-NMR spectrum is displayed in Fig 3A: all spectra were characterized by high reproducibility and consistency and no major variations among different samples could be identified. Raw data related to samples analyzed by ^1^H-NMR were reported in S1 Dataset. Selected buckets of the NOESY and CPMG spectra were analyzed by PCA (Fig 3B and 3C, respectively). Clusters enclosing samples of medium additives B (recalcified PPP), C (freeze and thaw, plasma depleted platelets) and D (freeze and thaw, PRP) were reciprocally segregated while the cluster related to samples of medium additive A (recalcified PRP) was scattered and partially overlapping with B and C.

**Fig 4:**
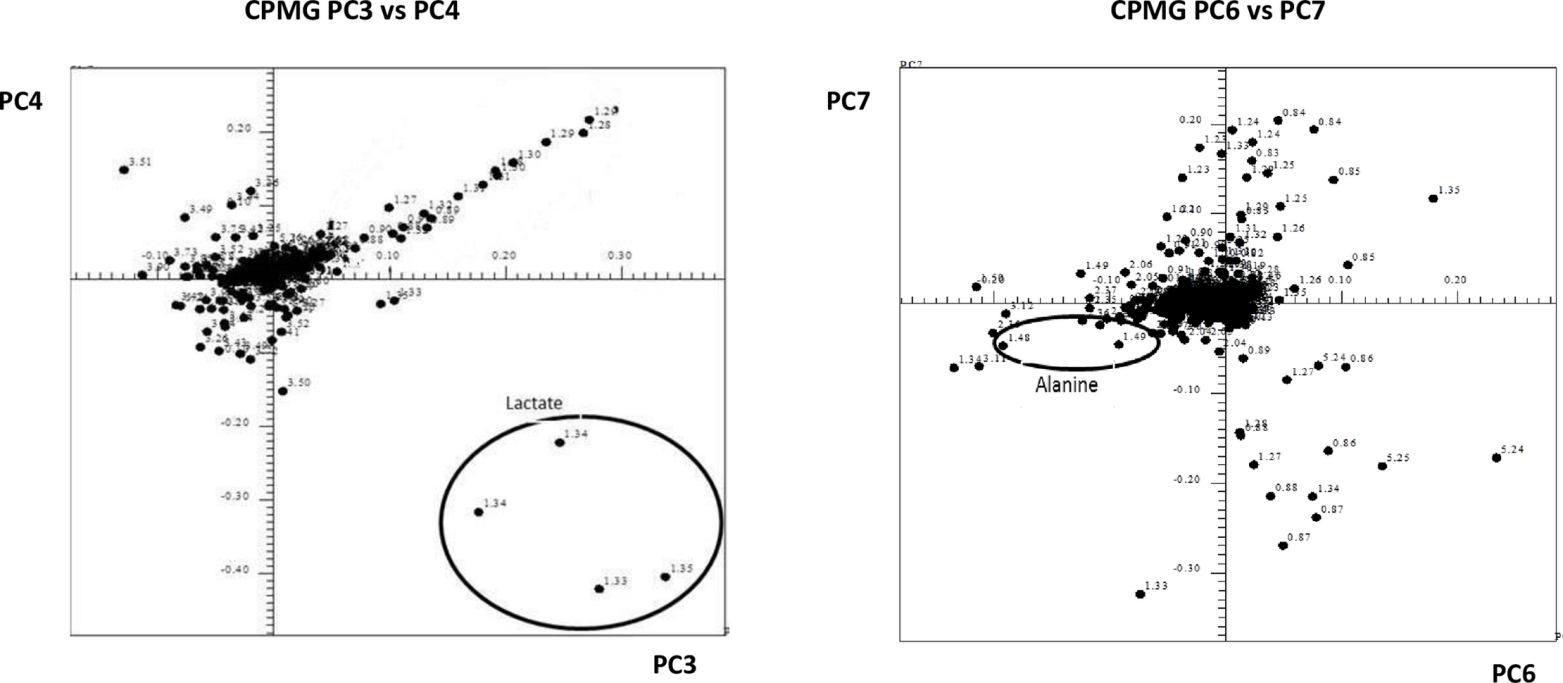
Bucketing analysis of CPMG spectra by PCA. PC3 *vs* PC4 signals as well as PC6 vs PC7 signals, respectively corresponding to lactate and to alanine are evidenced in the circles.

The bucket analysis of all NOESY and CPMG spectra (Fig 4) allowed identification of lactate and alanine signals as factors mostly contributing to the separation between medium additives, indicating that availability of these metabolites is different in the four products.

**Fig 5:**
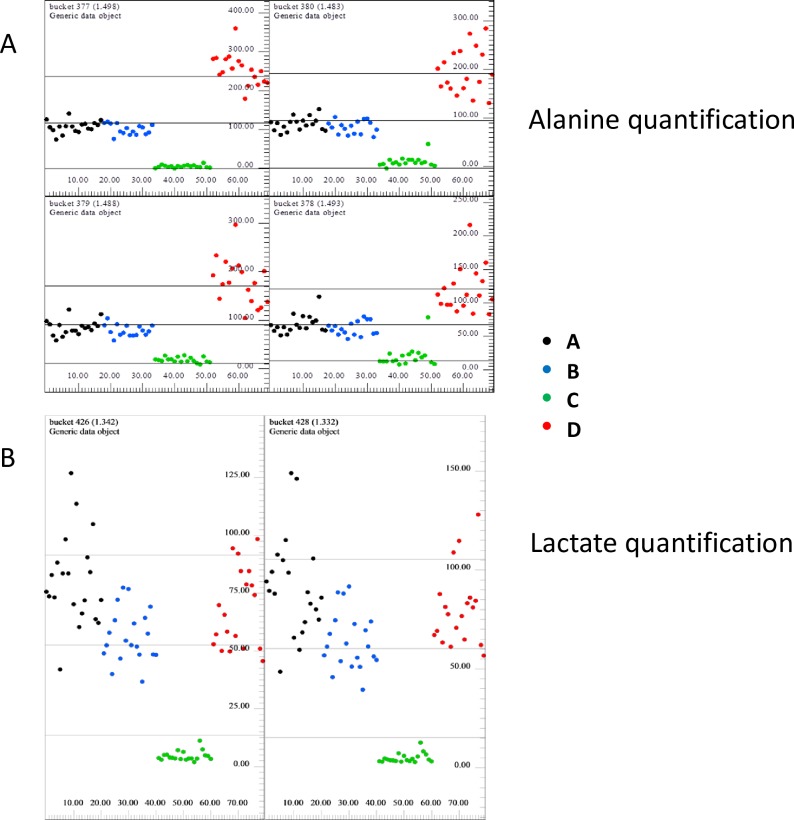
Relative quantification of alanine and lactate metabolites. (A) Bucket intensity of alanine signals (1.49 ppm). (B)shows bucket intensity of lactate signals (1.34 ppm).

Evaluation of bucket intensity provides also relative quantification of metabolites in the sample: results regarding alanine and lactate were reported in Fig 5A and 5B, respectively. Considering alanine quantification, the highest metabolite content could be found in samples of medium additive D (freeze and thaw, PRP); on the opposite, the lowest metabolite concentrations were measured in samples of medium additive C (freeze and thaw, plasma depleted platelets). Samples of A (recalcified PRP) and B (recalcified PPP) displayed intermediate concentration values. High lactate amounts were found in samples of the additive A, B and D, while extremely lower lactate amounts were measured in additive C samples.

### Resolution potential of MALDI-TOF MS

**Fig 6:**
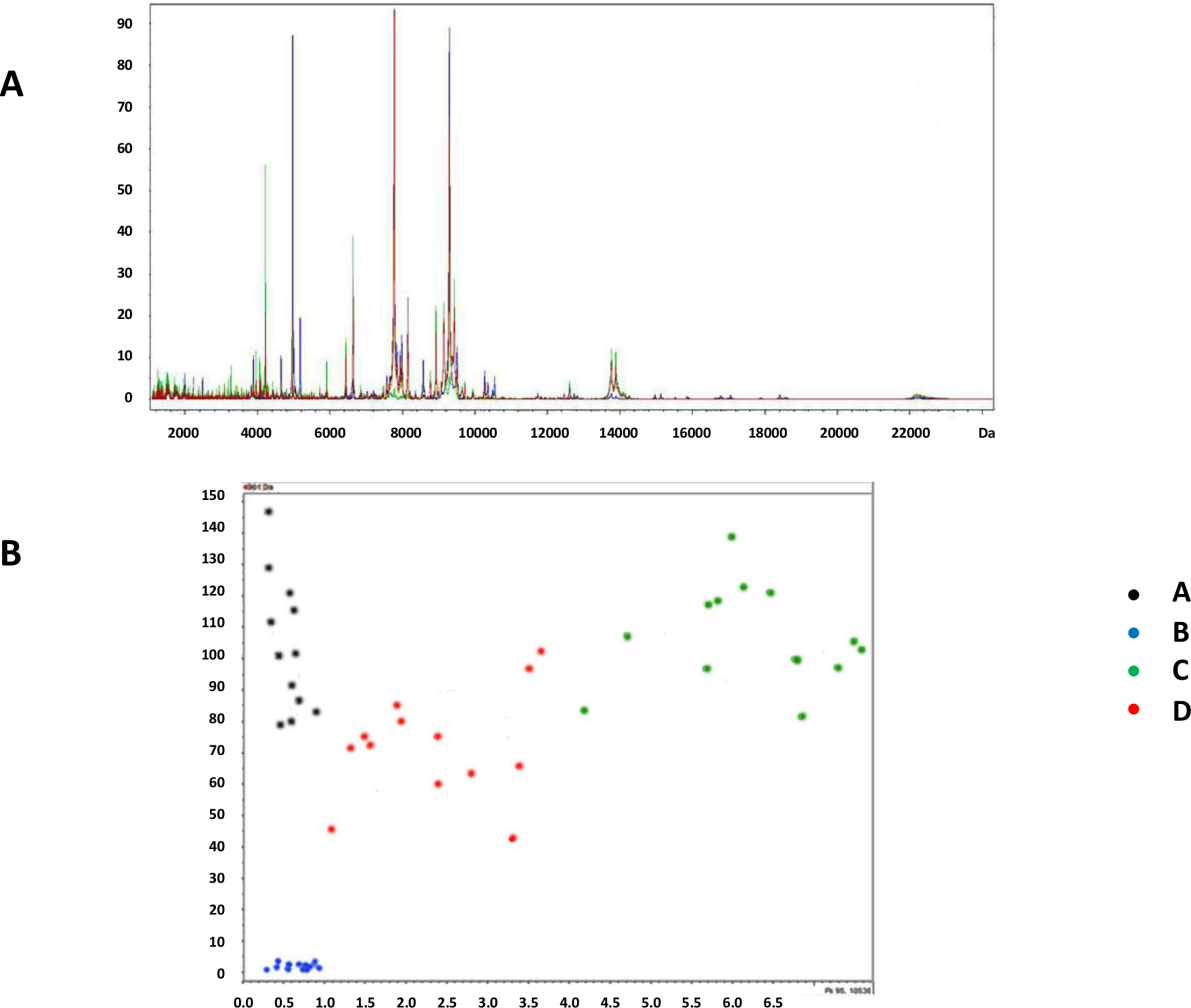
Sample analysis by MALDI-TOF MS. (A) Merged MALDI-TOF MS spectra obtained in all medium additive samples. (B) Visual separation of samples of the four medium additives A (recalcified PRP), B (recalcified PPP), C (freeze and thaw, plasma depleted platelets) and D (freeze and thaw, PRP) obtained by plotting areas of the two peaks with the highest contribution in the model created by Support Vector Machine algorithm. X-axis: 10,536 m/z peak area; y-axis: 4,961 m/z peak area.

Examples of MALDI-TOF MS spectra are displayed in Fig 6A and raw data related to samples analyzed by MALDI-TOF MS were reported in S2 Dataset. Selected peak areas of MALDI-TOF MS spectra were analyzed by 3 different classification algorithms: Genetic Algorithm, Quick Classifier and Support Vector Machine. The analysis took into account areas of the two peaks with the highest contribution in the model created by Support Vector Machine algorithm. Each statistic algorithm displayed a 100% capability to segregate samples of the different medium additives A (recalcified PRP), B (recalcified PPP), C (freeze and thaw, plasma depleted platelets) and D (freeze and thaw, PRP) in four separate and distinct groups (Fig 6B). Cross validation of sample assignation to each group was 97.1% for Quick Classifier, 98.1% for Genetic Algorithm and 100% for Support Vector Machine. The last model, therefore, was shown to efficiently display differences in the biochemical composition of the four medium additives.

## Discussion

ACT products are obtained after *ex vivo* cell expansion: GMP guidelines recommend to avoid the addition of animal derived products as FBS to promote cell proliferation *in vitro* [1]. Growth factors derived from human platelets can be used to obtain GMP compliant ACT products. Interindividual differences between platelet donors and medium additive manufacturing methods [10] can strongly affect the capability to stimulate cell growth *in vitro*. Especially when dealing with small scale manufactured medium additives for academic purposes, quality controls are needed to assess the capacity of the ancillary product to stimulate cell proliferation *in vitro* [9]. In this paper we aimed to define a rapid and cost effective test to potentially predict the impact of a medium additive on cell growth rate: this represents a significant achievement, improving both production and quality control processes. Applying four separate manufacturing protocols to PRP derived from a defined group of donors, we produced 4 kinds of medium additives as experimental model to perform a matched comparison regarding: a) their impact on *in vitro* cell growth; b) concentrations of selected growth factors, c) composition of ^1^H-NMR and MALDI-TOF MS spectra. We confirmed [10] that medium additives obtained from human platelet concentrates by distinct manufacturing protocols can differently affect cell proliferation rate. In particular, results obtained by the luminescence based cell proliferation assay demonstrated that medium additives A (recalcified PRP) and B (recalcified PPP) induced a faster ASC growth rate, when compared to additives obtained by freeze and thaw approach. Considering the manufacturing process, medium additive A is extremely close to the product we previously named SRGF [2,9]. SRGF used in this work was obtained from different aliquots of human PRP. Addition of 10% FBS to the cell culture medium was considered as a control condition: as expected considering previously published results [4,10], ASC expanded in presence of additives A and B were characterized by a higher proliferation rate when compared to 10% FBS. Elongated spindle shaped cells were previously demonstrated to be characterized by higher proliferation rate when compared to flat and round shaped cells [5,6]. Evaluating the ratio between cell axes we substantially confirmed that additives obtained through PRP recalcification induce higher ASC proliferation rate when compared to freeze and thaw derived ancillary products. In addition, we also confirmed that ASC expanded in presence of the platelet depleted medium additive B (recalcified PPP) were characterized by a lower growth rate when compared to medium additive A (recalcified PRP). Considering cell morphology, also ASC cultured in presence of medium additive A and B showed increased proliferation rate when compared to 10% FBS [4,10]. In synthesis, at least three out of the four investigated medium additives played a differential role on ASC proliferation rate in culture. Differences in the capability to stimulate cell proliferation between medium additive A and B were reasonably not detected by the luminescence based approach due to technical limitations of the assay. Other publications, in fact, demonstrated that growth factors extracted from PPP induced slower cell proliferation, when compared to platelet enriched mixtures [24]. Finally, considering medium additives obtained by freeze and thaw, we can suggest that, possibly due to the presence of residual plasma, solution C (freeze and thaw, plasma depleted platelets) could stimulate ASC proliferation to a similar extent when compared to additive D (freeze and thaw, PRP). Solution C, in fact was manufactured by a single platelet centrifugation step, followed by resuspension in PBS before platelet lysis by freeze and thaw procedure. This approach could leave a not-negligible quantity of plasma in the product. To reach the aims of this study we decided to manufacture separate medium additives sharing several biochemical features: thus, complete plasma removal, potentially leading to undesired platelet activation, was beyond our scopes. We acknowledge that, in principle, formulation of the different cell culture media should be normalized taking into account total protein amounts contained in each additive solution: nevertheless, considering the common and widespread approach applied in cell culture protocols, in this paper amounts of medium additives were defined and expressed as volume-to-volume fractions. In particular, we previously demonstrated that ASC and mesenchymal stem cells from other origin can be safely expanded with optimized growth rate in presence of 5% vol/vol SRGF [3,4].

After assessing the different cell proliferation promoting potential of our selected additives, we attempted to identify a reliable quality control test to unravel biochemical differences between such solutions. Growth factor concentration analysis is frequently used to characterize composition of platelet derived products with possible application as medium additives for cell expansion [2,12–15]. In this paper, we failed to identify representative concentrations of one or more specific growth factors as univocally characterizing each tested medium additive. Even performing PCA on concentration values of all growth factors measured in each single sample, we could not identify clearly separated clusters corresponding to the different medium additives. Despite minimal overlapping, only the group of samples of medium additive A (recalcified PRP) was spatially separated from the others in the graph: a reliable quality control test should be able to classify also the other ancillary products with lower performance *in vitro*. Otherwise, the comprehensive evaluation of the selected growth factor concentrations allowed only partial identification of medium additives differently regulating cell growth in culture: considering involved costs and technical burden we considered such result as not satisfactory. We acknowledge that measurements were performed on a reduced subset of samples: nevertheless, obtained data were in agreement with our previous publication [2] and we confirmed that concentration assessment of eight growth factors was not sufficient to classify differently obtained medium additives. In this work we chose singleplex ELISA kits to evaluate concentrations of growth factors as such analytic approach is normally applied for *in vitro* diagnostic purposes. Analytical properties of multiplex immunoassays are growing, but such high throughput and time convenient approach can be plagued by problems of cross reactivity of capture and detection antibodies [25]. For such reasons, both planar and suspension multiplex immunoassays are nowadays rarely used for diagnostic purposes. In this paper, we chose an analytic approach in compliance with diagnostic purposes because we aimed to identify innovative quality control tests to be applied in GMP regulated manufacturing processes. Analytic quantification of growth factor concentrations in commercially available FBS was not performed.

Therefore, we assayed the capability of ^1^H-NMR and MALDI-TOF MS techniques to unravel differences between selected medium additives we used to expand ASC. Such metabolomic approaches, in fact, allow a wide characterization of biological sample composition in a single, rapid and cost effective session of analysis. Metabolomic analysis of the commercially available FBS was not performed. In a previous publication [21], using a similar metabolomic approach, authors characterized biochemical alterations occurring during storage of human platelet apheresis products for transfusion. The analytical procedure allowed identification of distinct phases of platelet metabolic activation over time [21]. Applying appropriate statistical tools to the analysis of selected peaks obtained by ^1^H-NMR we could demonstrate that medium additives B (recalcified PPP), C (freeze and thaw, plasma depleted platelets) and D (freeze and thaw, PRP) can be distinguished between each other with good resolution. Nevertheless, samples of solution A (recalcified PRP) were not well separated from the others. This suggests that ^1^H-NMR can be a promising tool to estimate overall quality of platelet derived ancillary products in culture, but further efforts are required to improve the resolution capacity of such technique. We underline that, considering the production protocol, medium additive A is *de facto* what we previously named SRGF [2,9]. We demonstrated that such GMP compliant ancillary product can strongly stimulate growth rate of ASC in culture without affecting differentiation potential of such cells and avoiding transformation of cells after long term expansion [4]. As abovementioned, an appropriate quality control test should be able to better classify ancillary products characterized by different performances. Alanine relative quantification performed by ^1^H-NMR confirmed that such amino acid is contained in plasma and we can suggest that it is further and strongly released only after complete disruption of platelet membranes by freeze and thaw protocol. Lactate is known to be released by platelets: nevertheless, we suggest that the amount of released lactate is low, when compared to the relative availability of such metabolite in the plasma fraction contained in the apheresis product upon manipulation.

Beside ^1^H-NMR, we tested the analytic potential of MALDI-TOF MS. Adopting such approach all samples of each medium additive were reliably grouped in four well separated clusters: the resolution capacity was shown to be 100%. Thus, MALDI-TOF MS could identify, in a single analytical session, differences between medium additives characterized by strong and intermediate capacity to promote cell growth from other products with lower performance. When analyzing MALDI-TOF MS, spectra components significantly contributing to separation of medium additives were taken into account, but their specific characterization was not performed. Results of MALDI-TOF MS spectra analysis can represent a classification model to define the overall biological quality of a medium additive. In future applications, MALDI-TOF MS (and eventually also ^1^H-NMR) spectra analysis performed on a novel additive batch could allow its assignation to the category of products appropriately promoting proliferation (as A and B) or to the poorly performing ones (as C-D).

Beside peptidic growth factors, activated platelets can release other bioactive soluble mediators [26]. In particular, adenosine diphosphate [27] and thromboxane A2 [28] were shown to promote mesenchymal stem cell growth *in vitro*. Similarly, serotonin was shown to promote proliferation of endothelial and smooth muscle cells in culture [29,30]. While an elevate nitric oxide concentration was shown to reduce growth rate of primary cells [31,32], low nitric oxide doses promoted embryonic stem cell survival through apoptosis inhibition [33,34]. On the contrary, reactive oxygen species reduced cell viability and expansion rate due to induced cell membrane damage [35,36]. Finally, appropriate lipoprotein availability in the cell culture medium was shown to stimulate proliferation of human adult endothelial and smooth muscle cells [37]. As previously reviewed [38], MALDI-TOF MS was used to evaluate and characterize composition of the platelet peptidic secretome after activation of granule release [39]. Otherwise, ^1^H-NMR can better discriminate smaller metabolites released by platelets [40]. Nuclear magnetic resonance approaches allowed safe detection of serotonin and adenosine diphosphate released from platelet granules [41,42]. The same approach was shown to be suitable for the quantification of eicosanoids [43,44]. Moreover, electron paramagnetic resonance spectroscopy was demonstrated to permit the quantification of reactive oxygen species and of nitric oxide [45]. Finally, concentrations of different lipoprotein classes in human plasma could be assessed by ^1^H-NMR, coupled with an appropriate algorithm for data quantification [46,47]. Abovementioned evidences confirm that both ^1^H-NMR and MALDI-TOF MS can be useful techniques to classify and indirectly evaluate the capacity of a medium additive batch to stimulate ASC proliferation.

In summary, we here demonstrated that different experimental medium additives obtained from human platelets can diversely modulate ASC growth in culture. In parallel we showed that, evaluating a wide array or biological components, ^1^H-NMR and especially MALDI-TOF MS techniques can classify our medium additives in relation with their capacity to stimulate ASC growth *in vitro*. The identification of a quality control test predicting in a single assay the biological performance of a medium additive in culture can be considered as an important technical and procedural achievement. Considering GMP guidelines, we are aware that to fully standardize such quality control test, extensive technical, biological and statistical validation of the whole approach will be required.

## Conclusions

Considering the relatively low technical complexity and the high resolution potential of presently tested metabolomic approaches we suggest MALDI-TOF MS and, despite limitations, ^1^H-NMR as reliable and cost effective approaches to rapidly estimate the capacity of different medium additives to promote cell growth. The possibility to easily and rapidly select batches of appropriate medium additives with certified biological activity will markedly simplify the workflow to obtain ACT products with consistent production yields, as required by GMP guidelines.

## Supporting information

S1 File Raw luminescence data of ASC proliferation test. In this file we reported raw luminescence values collected to estimate ASC growth rate in presence of the different tested medium additives. As additional control, ASC growth rate was estimated also in presence of standard fetal bovine serum. Data analysis was reported beside raw values.
(XLSX)

S2 File Raw ASC major-to-minor-axis ratio data. In this file we reported major-to-minor-axis ratio that were collected to estimate ASC growth rate in presence of the different tested medium additives. Data analysis was reported beside raw values.
(XLSX)

S3 File Raw growth factor concentrations in the different medium additives. In this file we reported raw growth factor concentrations measured in analyzed samples of the different medium additives. Data analysis was reported beside raw values.
(XLSX)

S1 Dataset Raw ^1^H-NMR data. In this dataset we reported raw data related to 5 samples analyzed by ^1^H-NMR.
(ZIP)

S2 Dataset Raw MALDI-TOF MS data. In this dataset we reported raw data related to 5 samples analyzed by MALDI-TOF MS.
(ZIP)
